# Anthropometric measures and nutrition intake, habits and perceptions of Division I women’s volleyball players

**DOI:** 10.1186/1550-2783-8-S1-P8

**Published:** 2011-11-07

**Authors:** Danny Cortez, Laura Krebs-Holm, David Gish, Robert Wildman

**Affiliations:** 1 Food and Nutrition, School of Family & Consumer Sciences, Texas State University, San Marcos, TX 78666, USA; 2 Department of Health & Human Performance. Texas State University, San Marcos, TX 78666, USA

## Background

Volleyball is a physically demanding sport and success is based on aspects speed, power, agility, endurance, rapid processing and focus. Nutrition plays a significant role in maximizing performance and volleyball athletes need to be well-informed. Meanwhile, players can be self-conscious of body size and appearance especially in lieu of body contour revealing uniforms. At this time research-based information of this athletic population with regard to body composition, nutrition intake, habits and perceptions is limited and was studied.

## Methods

Twelve Division I women volleyball players aged 18.33±2.9 with 8.8±1.9 years of competitive volleyball experience participated in a study to assess body weight, composition and self-image as well as nutrition knowledge, perceptions, information resources and intake. Body composition was assessed using BOD POD (Life Measurement, Inc) and a 50-question survey was administered including questions addressing nutrition habits, perceptions and knowledge as well as self-image. Nutrition knowledge questions were validated and multiple choice while answers regarding nutrition and health perceptions and self-image were designed on a Likert scale and ranged from “Strongly Disagree” to “Strongly Agree”. Meanwhile a 3-day Food Journal was completed including two weekdays and one weekend day.

## Results

Results of anthropometric measures included height (176.2±7.4 cm), weight (73.3±6.8 kg), BMI (23.57±2.4), FM% (22.1±5.7%) and FFM% (77.9±5.7%). The average energy and protein intake was 1577±451 kcal/day and 1.04±0.23 g/kg with 52%, 28%, 20% of energy derived from carbohydrate protein and fat. The average intake of Vitamin C, B_1_, B_2_, B_3_, B_6_ B_12_ and zinc were above DRI recommendations while folate, calcium, iron and magnesium were below. Meanwhile 75% ofplayers alleged using one or more nutrition supplements ≥ 2 days/week. Only two of the players had taken a college nutrition course while seven indicated that they dedicated personal time to nutrition study and all ranked their coaches, friends and the internet as the primary sources of nutrition information. However, the players scored 38%±12% of the answers correct on a nutrition questionnaire while ranking water (hydration), protein and then carbohydrate in order of importance to maximizing sport performance. Related to health, 67% and 33% alleged never having their blood glucose and blood pressure and lipids checked. Furthermore, 75% either agreed or strongly agreed that they would like to change the way their body looks and worry about becoming fat while all players disagreed that skipping meals was a good way to control weight.

## Conclusion

In conclusion, the volleyball players assessed were lean on average and most were concerned about body weight and are calorie conscious and have a strong sense of self-image. Meanwhile, average energy intake was below estimated needs while energy distribution suggests emphasis on carbohydrate and protein food choices.

